# Cross-Sectional Study on Oral Nicotine Product Sales Trends in Scandinavia From 2018 to 2025

**DOI:** 10.2196/85490

**Published:** 2026-02-13

**Authors:** Marina A Murphy, Diane Henenberg, Lindsay Reese

**Affiliations:** 1 HAYPP Limited, 33 Clarke Road, Mount Farm, Milton Keynes, MK1 1LG, United Kingdom, 44 07971519849; 2 Snusbolaget AB, Stockholm, Sweden

**Keywords:** Norway, Sweden, market research, sales, nicotine pouch, snus, harm reduction

## Abstract

This cross-sectional analysis of more than 19 million e-commerce orders from Sweden and Norway indicates that nicotine pouches have overtaken traditional snus in market share in both countries, reinforcing the potential of nicotine pouches as a harm reduction tool.

## Introduction

Nicotine pouches (NPs) are rapidly gaining market traction, particularly in countries with established smokeless tobacco use. They differ from snus, a widely used smokeless tobacco product in Scandinavia, in that they do not contain any tobacco leaf. Chemical analyses confirmed that NPs contain substantially fewer and lower levels of toxicants compared with snus, with toxicant profiles similar to pharmaceutical nicotine replacement therapies [1]. Nicotine is dependence forming and should not be used by vulnerable populations such as youth, pregnant women, and individuals with certain health conditions; however, for adults who choose to use nicotine, NPs are increasingly popular. The United States is the largest NP market, and emerging evidence suggests that daily NP use is most common among adults who recently quit another product and rare among people who never used nicotine [2]. This encouraging trend suggests that NPs—originally designed as a harm reduction tool [3]—are indeed displacing more harmful products. This study examines whether a similar pattern is emerging in two Nordic countries where snus has traditionally been the dominant tobacco product.

## Methods

### Study Design

This cross-sectional study analyzed HAYPP Group AB (Stockholm, Sweden) sales data from January 1, 2018, to September 17, 2025, on 19,528,087 purchases of snus and NPs by 1,721,752 customers from seven e-commerce websites in two countries (Sweden: snusbolaget.se, haypp.se, nettotobak.se, and snusnetto.se; Norway: snuslageret.no, snushjem.no, and snus.com). In both countries, purchases are age-verified with government-issued identification numbers that encode each customer’s birth date and gender. This information is authenticated by payment service providers. After anonymization (permanently removing all identifiers), aggregated data were used to calculate volume shares by product and by gender. The number of cans of NPs or snus sold in each year was divided by the total number of cans of both products sold in each country.

### Ethical Considerations

Under Swedish law (Ethical Review Act, SFS 2003:460 [4]), ethical approval is required for research involving physical intervention on humans, biological material traceable to individuals, or processing of sensitive personal data. This study analyzed fully anonymized, aggregated sales data without any personal identifiers or direct human participation. Therefore, it falls outside the scope of the Act and was not submitted for ethical review. Under the European Union (EU) General Data Protection Regulation (GDPR 2016/679 [5]), ethical approval was not required for this market research study because it used fully anonymized sales data, and no personal data or direct human participation was involved. The study complies with the International Chamber of Commerce/European Society for Opinion and Market Research (ICC/ESOMAR) International Code on Market, Opinion and Social Research, and Data Analytics [6].

All customers consented to use of their anonymized, aggregated data when reviewing and accepting the privacy policy [7]. No compensation was provided for the use of these sales data.

## Results

**Figure 1.**
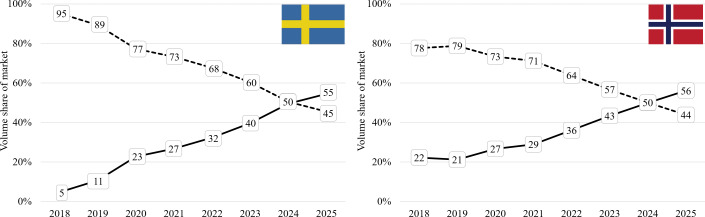
Cross-sectional volume shares of the oral nicotine product markets (2018‐2025) for nicotine pouches (NP, solid lines) and snus (dotted lines) in Sweden (left) and Norway (right).

The sales data cover 13,995,343 and 5,532,744 individual orders from 1,253,066 and 468,686 unique customers in Sweden and Norway, respectively. NPs surpassed snus in market share in 2025 in both countries (Figure 1). The Swedish NP volume share rose from 5% in 2018 to 55% in 2025, while snus declined from 95% to 45%. In Norway, the NP share rose from 22% to 56% over the same period, with snus declining from 78% to 44%.

**Figure 2.**
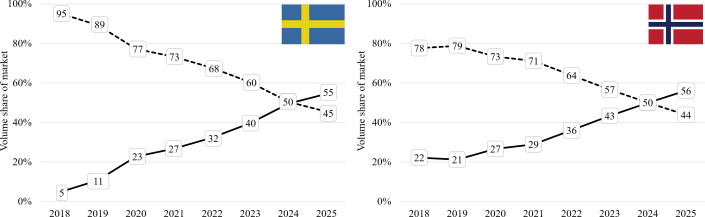
Cross-sectional volume shares of the oral nicotine product markets (2018‐2025) for men (blue) and women (red) for nicotine pouches (NP, solid lines) and snus (dotted lines) in Sweden (left) and Norway (right).

Women first purchased more NPs than snus in 2022 (Figure 2) due to their lower 2018 market shares of snus (~25%). Men still purchase more snus than NPs in both countries, but this could change in 2026 if trends persist.

## Discussion

This descriptive analysis is the first cross-country comparison of NP and snus sales trends in Scandinavia. Data from more than 19 million e-commerce orders show that NPs overtook snus in the market share in Sweden and Norway in 2025, offering early evidence of market displacement in both countries. Taken together with US findings [2], these results suggest that NPs could be reshaping nicotine consumption in ways aligned with tobacco harm reduction, although ongoing monitoring and regulation will be critical to maximize benefits and minimize risks.

Although these observations do not reflect individual-level use, they highlight important shifts in consumer behavior that mirror the original “Swedish experience” where 50 years ago, men shifted from cigarettes to snus [8], and daily smoking decreased from 40% to 15% between 1976 and 2002 [9]. Over the same time frame, daily smoking rates for Swedish women dropped from 34% to 20% [9]. A similar trend materialized in Norway after low-nitrosamine snus was introduced in the 1990s, with men moving away from cigarettes before women [10,11]. The EU banned snus in 1992, but Sweden obtained an exemption. Norway is not part of the EU. Long-term epidemiological data indicate that this shift offers a substantial harm reduction benefit, as evidenced by low incidences of tobacco-related disease in Sweden compared to the rest of the EU [12].

The US Food & Drug Administration recognizes snus as a lower-risk alternative to cigarettes, authorizing eight products with the claim: “Using General Snus instead of cigarettes puts you at a lower risk of mouth cancer, heart disease, lung cancer, stroke, emphysema, and chronic bronchitis” [13]. NPs contain even fewer toxicants [1], placing them at the lowest risk end of the non-medicinal tobacco and nicotine product continuum. Maximizing public health would require encouraging switching from higher-risk products to lower-risk products such as NPs, clear regulation, youth access prevention, and efforts to support cessation among current users who would like to stop using nicotine.

A limitation of this study is its reliance on sales data from a single e-commerce company. However, these platforms account for considerable snus and NP market shares in both countries. National surveys currently lack comparable precision. Statistik Sentrabyrå (Norway) does not distinguish between snus and NPs, and Folkhälsomyndigheten (Sweden) only began differentiating between snus and NPs in 2022. While future surveys will provide more nuanced nationally representative information, these market data likely offer a more accurate and timelier picture of an evolving Nordic nicotine landscape. Future research should explore user transitions across cigarettes, snus, NPs, and cessation, alongside long-term health and policy effects.

## Abbreviations

EU: European Union
NP: nicotine pouch
