# Correction: Cross-Sectional Study on Oral Nicotine Product Sales Trends in Scandinavia From 2018 to 2025

**DOI:** 10.2196/94157

**Published:** 2026-03-05

**Authors:** Marina A Murphy, Diane Henenberg, Lindsay Reese

**Affiliations:** 1 HAYPP Limited, 33 Clarke Road, Mount Farm, Milton Keynes, MK1 1LG, United Kingdom, 44 07971519849; 2 Snusbolaget AB, Stockholm, Sweden

In “Cross-Sectional Study on Oral Nicotine Product Sales Trends in Scandinavia From 2018 to 2025” [1], the authors noted one error.

In the original published paper, the Figure 1 image was erroneously duplicated for Figure 2. Figure 2 has been updated to the following image (attached here as Figure 1):

**Figure 1.**
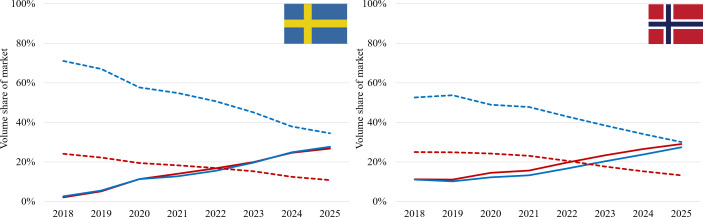
New Figure 2: Cross-sectional volume shares of the oral nicotine product markets (2018‐2025) for men (blue) and women (red) for nicotine pouches (NP, solid lines) and snus (dotted lines) in Sweden (left) and Norway (right).

The correction will appear in the online version of the paper on the JMIR Publications website, together with the publication of this correction notice. Because this was made after submission to PubMed, PubMed Central, and other full-text repositories, the corrected article has also been resubmitted to those repositories.
